# Supplementary material for: Nuclear Reorganization in Hippocampal Granule Cell Neurons from a Mouse Model of Down Syndrome: Changes in Chromatin Configuration, Nucleoli and Cajal Bodies
Source: Int J Mol Sci. 2021 Jan 27;22(3):1259. doi: 10.3390/ijms22031259 (PMC7865916; doi:10.3390/ijms22031259)
Supplement: Supplementary file 1 [file ijms-22-01259-s001.pdf]

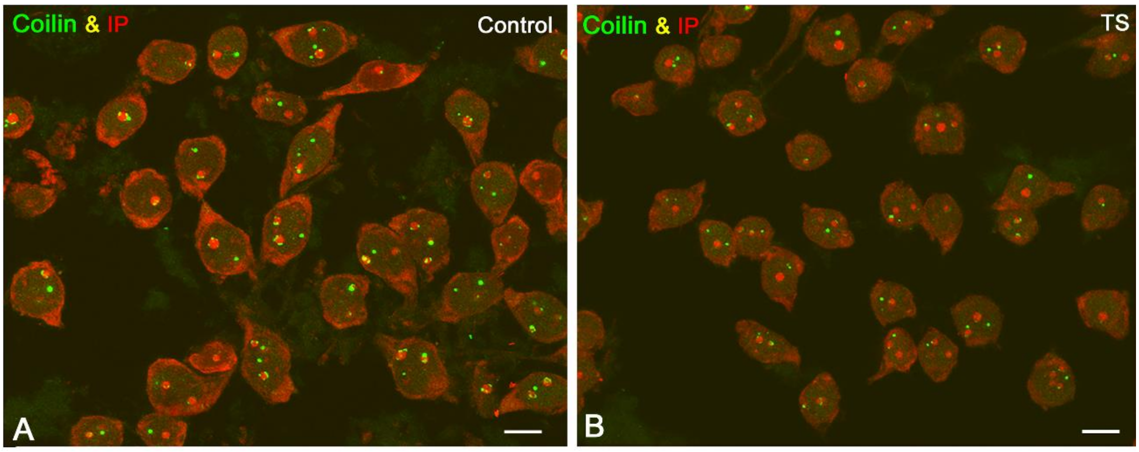

Figure S1

Supplementary Figure S1. Representative example of dissociated dentate gyrus GCs from control and TS mice immunostained for coilin and co-stained with propidium iodide (PI), a cytochemical staining for RNAs. Note the higher RNA staining intensity in the cytoplasmic band of control GCs as compared with TS neurons. Moreover, these confocal images clearly illustrate the apparent reduction of CBs in TS GCs relative to control ones. Scale bar: 10μm.

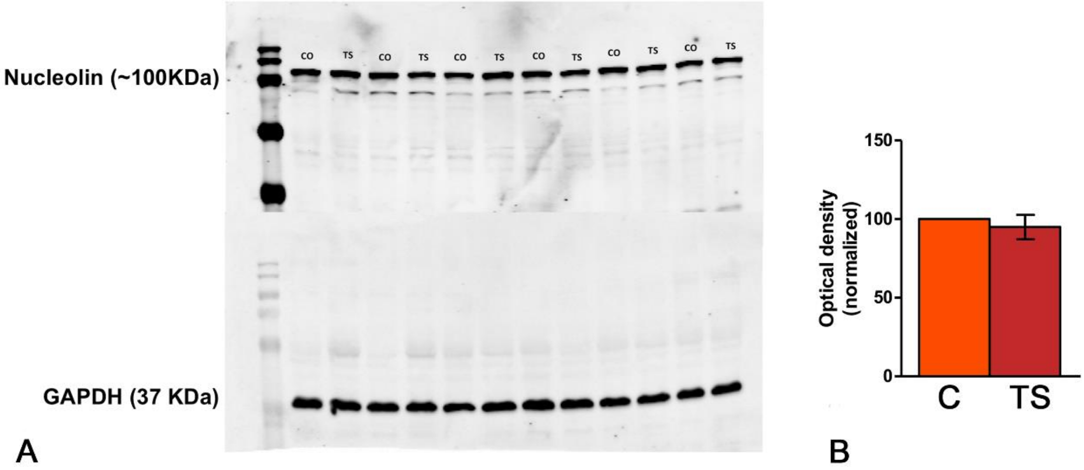

Figure S2

Supplementary Figure S2: Representative image (A) and analyses (B) of the western blot showing no differences in the expression of nucleolin in the hippocampi of TS mice relative to control (CO) mice. GAPDH was used as a loading control. Six independent animals of each genotype were used in the experiment. CO = control mice; TS = Ts65Dn mice.
